# miR-708-5p and miR-34c-5p are involved in nNOS regulation in dystrophic context

**DOI:** 10.1186/s13395-018-0161-2

**Published:** 2018-04-27

**Authors:** Marine Guilbaud, Christel Gentil, Cécile Peccate, Elena Gargaun, Isabelle Holtzmann, Carole Gruszczynski, Sestina Falcone, Kamel Mamchaoui, Rabah Ben Yaou, France Leturcq, Laurence Jeanson-Leh, France Piétri-Rouxel

**Affiliations:** 1 Sorbonne Université-UMRS974-Inserm-Institut de Myologie, 105 bd de l’Hôpital, 75013 Paris, France; 2 AP-HP, Centre de Référence Maladies Neuromusculaire Nord, Est, Ile-de-France, G.H. Pitié-Salpêtrière, F-75013 Paris, France; 3 Laboratoire de Génétique et Biologie Moléculaire, Hôpital Cochin, Paris, France; 4 Généthon, 1 rue de l’Internationale, 91000 Evry, France

**Keywords:** Duchenne muscular dystrophy (DMD), Becker muscular dystrophy (BMD), miRNA, nNOS

## Abstract

**Background:**

Duchenne (DMD) and Becker (BMD) muscular dystrophies are caused by mutations in the *DMD* gene coding for dystrophin, a protein being part of a large sarcolemmal protein scaffold that includes the neuronal nitric oxide synthase (nNOS). The nNOS was shown to play critical roles in a variety of muscle functions and alterations of its expression and location in dystrophic muscle fiber leads to an increase of the muscle fatigability. We previously revealed a decrease of nNOS expression in BMD patients all presenting a deletion of exons 45 to 55 in the *DMD* gene (BMDd45-55), impacting the nNOS binding site of dystrophin. Since several studies showed deregulation of microRNAs (miRNAs) in dystrophinopathies, we focused on miRNAs that could target nNOS in dystrophic context.

**Methods:**

By a screening of 617 miRNAs in BMDd45-55 muscular biopsies using TLDA and an in silico study to determine which one could target nNOS, we selected four miRNAs. In order to select those that targeted a sequence of 3′UTR of *NOS1*, we performed luciferase gene reporter assay in HEK393T cells. Finally, expression of candidate miRNAs was modulated in control and DMD human myoblasts (DMDd45-52) to study their ability to target nNOS.

**Results:**

TLDA assay and the in silico study allowed us to select four miRNAs overexpressed in muscle biopsies of BMDd45-55 compared to controls. Among them, only the overexpression of miR-31, miR-708, and miR-34c led to a decrease of luciferase activity in an *NOS1*-3′UTR-luciferase assay, confirming their interaction with the *NOS1*-3′UTR. The effect of these three miRNAs was investigated on control and DMDd45-52 myoblasts. First, we showed a decrease of nNOS expression when miR-708 or miR-34c were overexpressed in control myoblasts. We then confirmed that DMDd45-52 cells displayed an endogenous increased of miR-31, miR-708, and miR-34c and a decreased of nNOS expression, the same characteristics observed in BMDd45-55 biopsies. In DMDd45-52 cells, we demonstrated that the inhibition of miR-708 and miR-34c increased nNOS expression, confirming that both miRNAs can modulate nNOS expression in human myoblasts.

**Conclusion:**

These results strongly suggest that miR-708 and miR-34c, overexpressed in dystrophic context, are new actors involved in the regulation of nNOS expression in dystrophic muscle.

**Electronic supplementary material:**

The online version of this article (10.1186/s13395-018-0161-2) contains supplementary material, which is available to authorized users.

## Background

Duchenne muscular dystrophy (DMD) is a progressive and fatal X-linked neuromuscular disorder caused by mutations in dystrophin gene (*DMD*) [1, 2]. The disease is due to mutations that disrupt the translational reading frame, leading to the loss of the protein dystrophin expression. Mutations in the *DMD* gene that preserve the open reading frame allow the production of an abnormal truncated dystrophin still retaining some functional capacity, leading to a milder muscle disease (Becker muscular dystrophy or BMD) [3, 4]. This feature is the rationale of exon skipping therapy and genome editing strategies now in development [5–7]. The principle of these approaches is to delete one or multiple exons in order to obtain the production of a truncated dystrophin, inducing a phenotypic conversion of DMD into BMD. To apply these strategies to a larger number of eligible patients, the skipping of exons 45 to 55 of the *DMD* gene has been proposed since that could correct the reading frame in about 63% of DMD patients with deletions [8, 9]. Given the perspective of this approach, the therapeutic relevance of the resulting 45–55 truncated dystrophin may be deduced from the clinical status of BMD patients carrying spontaneous deletion of exons 45 to 55 of the *DMD* gene (BMDd45-55). Likewise, molecular investigations of factors involved in pathophysiological process occurring in muscle of these patients are of great interest.

Dystrophin is a 427-kDa protein that links the cytoskeleton to sarcolemma via the dystrophin-associated protein complex (DAPC) [10]. DAPC provides stability and integrity to the muscle membrane during contraction. The loss of dystrophin leads to a breakdown of the DAPC complex, and as consequences, the muscle fibers become more sensitive to mechanical stresses, leading to muscle degeneration, chronic inflammation, or increased fibrosis [11, 12]. Among the partners of the dystrophin, the neuronal nitric oxide synthase (nNOS), that synthesizes nitric oxide (NO), was shown to play critical roles in a variety of muscle functions, including not only contraction, regeneration, atrophy, glucose uptake, and blood perfusion [13] but also transcriptional regulation [14]. Indeed, NOS enzymatic activity was recently demonstrated as essential for the rescue of muscle mass after atrophy induced by unloading [15], as well as in reducing the extent of atrophy during disease [16], and these effects were mostly assigned to activation of muscle stem cells by the NO production. Three different isoforms of nNOS, namely nNOSα, nNOSβ, and nNOSμ, were described to be expressed in the skeletal muscle. The nNOSμ, the major one, contains a PDZ domain which allows its binding to the rod domain of the dystrophin at the spectrin-like repeats 16 and 17 (R16/17) encoded by exons 42–45 [17]. It has been shown that in the absence of dystrophin, nNOSμ was delocalized from the sarcolemma of the muscular fibers and its expression decreased [18, 19]. Alteration of nNOSμ expression and location was described to contribute to DMD pathophysiology by the disturbance of NO signaling leading to an increase of the muscle fatigability [20, 21].

Our goal was to identify the molecular factors that can modulate the expression of nNOS and the muscular biopsies of BMDd45-55 patients that were sought to be a pertinent tool. Indeed, in these patients, the deletion of the 45–55 exons in dystrophin mRNA should partially delete the spectrin motif repeat 17 in the resulting protein and could alter the nNOSμ anchoring. A previous study revealed that the BMDd45-55 patients displayed variable clinical and histological phenotypes and that a subsequent decrease of nNOS protein expression occurred in these patients compared to healthy subjects [22]. Furthermore, several studies demonstrated a deregulation of miRNA expression profiles in dystrophinopathies [14, 23–26]. Cacchiarelli et al.’s study showed also that the loss of nNOS sarcolemmal localization leads to the deregulation of the expression of several microRNAs (miRNAs) [14]. miRNAs are short noncoding RNA that regulate mRNA post-transcriptionally either by promoting mRNA degradation or by inhibiting protein translation [27]. miRNAs have been shown to regulate functions of the skeletal muscle both in normal and pathological states [14, 28–30]. Altogether, these studies suggest a link between miRNA expression, nNOS expression, and physiopathology of dystrophinopathies. Thus, the aim of the present study was to identify miRNAs that could modulate nNOS expression by screening the miRNA profile in BMDd45-55 muscular biopsies.

## Methods

### Ethics approvals

Muscle biopsies were collected from patients after informed consent, and this study was performed with agreement from the Committee for the Protection of Persons (CPP) concerned.

### Cohort of patients

Nine Becker muscular dystrophy (BMD) patients characterized for a deletion of exons 45–55 of the *DMD* gene were studied. These patients were already described [22]. Indeed, the clinical status of the patients was scored using the Gardner–Medwin and Walton scale (GMWS) [31], and the histopathological status based on routine hematoxylin and eosin (HE) staining of muscle cryosections has been investigated showing a large histological disparity. These criteria allowed defining three classes of severity: (i) “mild” (GMWS ≤ 2 [i.e., still able to normally climb stairs] and normal muscle biopsy); (ii) “moderate” (GMWS ≤ 2 or mild dystrophic muscle biopsy); (iii) “severe” + (GMWS ≤ 2 and/or dystrophic muscle biopsy) (Table 1). The 9 patients were biopsied at an age ranging from 9 to 69 years old for diagnostic purposes after informed consent, and these biopsies were used during the experiments in the present article. In addition, the five muscle biopsies used as healthy control muscles (Ctrl) were recovered as surgical wastes from orthopedic surgery of individuals without neuromuscular diseases. All human muscle biopsies were flash frozen in isopentane cooled in liquid nitrogen and evaluated for dystrophin and nNOS expression by Western blotting [22].

**Table 1:** Clinical and histopathological phenotypes compared with nNOSμ expression in BMDd45-55 patients

Name	Severity class	Age at muscle biopsy	Histopathological status (*)	nNOSμ protein expression (**)
Ctrl 1		26	N	**++**
Ctrl 2		40	N	**++**
Ctrl 3		10	N	**++**
Ctrl 4			N	**++**
Ctrl 5			N	**++**
Patients				
BMD 1	Moderate	35	**+/−** (35)	**+/−**
BMD 2	Severe +	13	**+** (13)	**+**
BMD 4	Severe +	33	**++** (33)	**+**
BMD 7	Moderate	40	**+/−** (40)	**+**
BMD 8	Moderate	12	**+/−** (12)	**+/−**
BMD 11	Mild	69	N (69)	**+**
BMD 12	Moderate	18	**+/−** (18)	**+/−**
BMD 18	ND			
BMD 31	ND			

(*) N normal, +/− mild dystrophy, *+ and ++* severe dystrophy, *ND* not determined, (**) +/− traces or not detectable in Western blot, + weak, ++ normal

### Taqman Low-Density Array (TLDA)

Total RNA (including miRNA and mRNA) were extracted from about 30 mg of muscular biopsy using the NucleoSpin© miRNA kit from Macherey-Nagel. Total RNA (200 ng) was reverse-transcribed with the Megaplex Primer Pools A and B (human version 3), and miRNAs were quantified after a pre-amplification step, with TaqMan Array MicroRNA Cards A and B (human version 3) on the 7900HT Real-Time PCR System (AB) according to the manufacturer’s guidelines. Relative quantification was performed with 2^−dCT^ method, using the mean of all miRNA expressed as normalizer.

### miR-target predictions

The ability of candidate miRNAs to target *NOS1*-3′UTR was evaluated with Diana-microT algorithm and TargetScan v6.2.

### Individual RT-qPCR

Thirty milligrams of muscular biopsies from 3 healthy subjects and patients BMD1, 2, 4, 8, and 11 was extracted using the NucleoSpin© miRNA kit (Macherey-Nagel). One hundred nanograms of RNA was reverse-transcribed with Universal cDNA synthesis kit II (Exiqon) according to the manufacturer’s instructions. Complementary DNAs (cDNAs) were analyzed by real-time quantitative PCR performed on Light Cycler 480 instrument (Roche) using Exilent SybR Green Master Mix (Exiqon). LNA™ PCR Primers set from Exiqon were used for miRNA expression analysis (miR-212: 204170, miR-708: 204490, miR-34c: 205659, miR-31: 204236). miRNA expression was normalized on miR-30b-5p expression (204765) using 2^−dCT^ method.

### Luciferase assay

Genomic DNA was extracted from biopsies of healthy subject using NucleoSpin© Tissue kit from Macherey-Nagel, following the manufacturer’s instructions. gDNA was eluted after incubation of the silica membrane 3 min, twice in 50 μL of Elution Buffer BE, followed by centrifugation 1 min at 11,000*g*, being 100 μl of total gDNA.

*NOS1-*3′UTR (Ensembl: ENST00000618760) was cloned downstream of Firefly luciferase gene in HSVTK-Luc3′ modified plasmid. As this 3′UTR is 7183 pb in length, it is too large to be fully cloned in this plasmid. Therefore, we cut it in 4 overlapping parts using following primers that add restriction sites (in bold) on gDNA fragments (Table 2).

**Table 2:** Primers used to fragment NOS1-3′UTR

Position in 3′UTR)	Forward primer	Reverse primer
Part 1 (1-1896)	F1-5′TTG**TCTAGA**CTGGACCCTCTTGCCCAGC-3′	R1-5′AAG**GATATC**CAGGGGAAATTGGGATTAAAGG-3′
Part 2 (1773-3685)	F2-5′-AAC**TCTAGA**CTATGACTCACCTTGCTCTGC-3′	R2-5′-ATC**GATATC**CTTACATGCTCCCTGTCCGTG-3′
Part 3 (3607-5523)	F3-5′-AAT**TCTAGA**CTGGTAGCTTCTGGAAGGTAAG-3′	R3-5′AAT**GATATC**GCCACAAGGCAGGGACTGGC-3′
Part 4 (5358-7149)	F4-5′-TAG**TCTAGA**GAAACACAGGTCTGAGGGTCTG-3′	R4-5′-CC**GATATC**ATTGTAACCATAATGCAAACAAGC-3′

Added restriction sites are indicated in bold characters (XbaI and EcorV)

Fragments were amplified using Mastermix Phusion with the following protocol: 98 °C 30s; 10 cycles of 98 °C 10s, 58 °C 30s, and 72 °C 1 min; and 20 cycles of 98 °C 10s, 61 °C 30s, 72 °C 1 min, and 72 °C 10 min.

Amplicons were purified with NucleoSpin© Gel and PCR cleanup from Macherey-Nagel. 3′UTR parts were then cloned in HSVTK-Luc3′ modified plasmid between XbaI and EcorV sites.

Each 3′UTR construction (24.5 ng) was co-transfected in 293T-HEK cells with 25 pg of either miR-negative control (AM17111, Ambion) or miR-212, miR-31, miR-34c, or miR-708 (AM17100, Ambion) using lipofectamine 2000 diluted in Optimem reduced medium. The plasmid CMV-Renilla luciferase (0.25 ng) was also transfected in each condition as normalizer. Five hours post-transfection, Optimem reduced medium is replaced with DMEM added with FBS 10%. Twenty-four hours after transfection Firefly and Renilla luciferase luminescences were quantified with Dual-Glo Luciferase Assay System (according to the manufacturer’s instructions) on Flexstation 3 Microplate reader. Firefly luciferase activity was normalized on Renilla luciferase activity.

### Human myoblast transfection

Human immortalized myoblasts from a healthy subject (ctrl) and from a DMDd45-52 patient (DMDd45-52) were used [32]. Myoblasts were plated 48 h before transfection at 3 × 10^4^/well of 6-well plate or 6 × 10^3^/well of 24-well plate in proliferation medium composed of DMEM supplemented with 5 μg/ml of insulin, 5 ng/ml of EGF, 0.5 ng/ml of bFGF, 0.2 μg/ml of dexamethasone, 25 μg/ml of fetuin, 20% of fetal bovine serum, and 16% of medium 199. Cells were transfected with 12.5 pg of either miR-negative control (AM17111, Ambion); miR-31, miR-34c, or miR-708 (AM17100, Ambion); or antimiR-34c or antimiR-708 (AM17000, Ambion) using lipofectamine 2000 diluted in Optimem reduced medium. Twenty-four hours after transfection, transfection medium was replaced with proliferation medium for 24 h.

### miRNA expression

Cells were harvested in 300 μl of Buffer ML (NucleoSpin© miRNA, Macherey-Nagel). Total RNA (small + large RNA) was extracted from lysed cells with NucleoSpin© miRNA kit (Macherey-Nagel) following the manufacturer’s instructions. cDNA generated with Universal cDNA synthesis kit II (Exiqon) according to the manufacturer’s instructions was analyzed by real-time quantitative PCR performed on Light Cycler® 480 instrument (Roche) using Exilent SybR Green Master Mix (Exiqon). LNA™ PCR Primers set from Exiqon were used for miRNA expression analysis (miR-708: 204490, miR-34c: 205659, miR-31: 204236). miRNA expression was normalized on SNORD44 expression (203902) using 2^−dCT^ method.

### Western blotting

For nNOS detection, cells were lysed in 50 μl of RIPA buffer (150 mM NaCl,50 mM 4-(2-hydroxyethyl)-1-piperazineethanesulfonic acid, pH 7.4, 5 mM ethylene diamine tetra acetic acid, 1% NP-40, 0.5% sodium deoxycholate, 0.1% sodium dodecyl sulfate, 1 mM PMSF with a mix of protease inhibitors (Roche), and centrifuged 10 min 1500*g* at 4 °C.

Protein extracts (20 μg) were denaturated in Laemmli buffer 2× added of 10% of 2-mercaptoethanol 30 min at room temperature (RT) and incubated 15 min in ice and then 15 min at RT. Proteins were resolved by SDS–PAGE (4–12%, Invitrogen) and transferred to nitrocellulose. Membranes were blocked in tris-buffered saline 0.1% Tween-20 with 5% non-fat dry milk 1 h at RT and incubated overnight at 4 °C, with rabbit polyclonal nNOS antibody (R-20, Santa Cruz, 1:100) or with mouse monoclonal GAPDH antibody (MAB9748, Tebu-Bio, 1:8000). After being washed in TBS 0.1% Tween, membranes were incubated for 1 h at RT with secondary antibodies: goat anti-rabbit-horseradish peroxidase (HRP) (1/50000) or sheep anti-mouse HRP (1/15000) (Jackson Immunoresearch). Western blots were revealed with enhanced chemiluminescence (Thermo Scientific) with Image Quant LAS 4000 system (GE Healthcare Life Sciences).

### Immunostaining

Cells in 24-well plate were washed with PBS and fixed with paraformaldehyde 4% 10 min at RT and washed 3 times in PBS. Fixed cells were permeabilized with 0.5% Triton X-100 (Sigma-Aldrich), washed, and blocked in PBS/5% bovine serum albumin (BSA) for 40 min at RT. Cells were then incubated in PBS/1% BSA/0.1% saponin with a goat polyclonal anti-nNOS antibody (Ab1376, Abcam, 1:500), overnight at RT; washed in PBS/1%BSA/0.1% saponin; and incubated for 1 h with secondary antibody: Donkey anti-goat (Alexafluor 594 conjugate, Life Technologies, 1:500) and with DAPI (1:5000, Sigma). Fixed cells were then thoroughly washed in PBS/1% BSA/0.1% saponin and then in PBS and mounted in Fluoromount (Southern Biotech). Images were acquired with Leica DM2500 confocal microscope using × 63 objective.

### Statistical analysis

Statistical analysis were performed using Student’s *t* test. A value of *p* < 0.05 was considered statistically significant.

Methods used for the Additional file 4: Figure S1 are described in Additional file 1.

## Results

### miRNA expression profiling in BMDd45-55 muscular biopsies

**Fig. 1:**
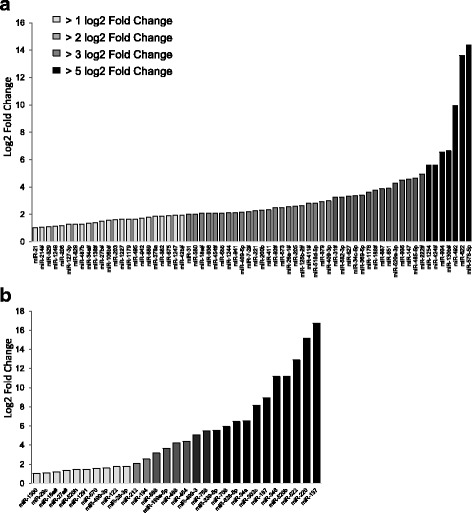
Screening of miRNA expression profiling by TLDA in BMDd45-55 muscular biopsies. Data of TLDA were expressed by the value of Log2(R), where *R* is the ratio of the average of the relative quantification (RQ) obtained in BMDd45-55 muscles on the average of RQ values obtained from muscle of healthy subjects (**a**), or obtained from muscle of severe BMDd45-55 patients on the average of RQ values obtained from muscle of the moderate and mild BMDd45-55 patients (**b**), *p* ≤ 0.05. RQ are obtained using average of values of all miRNA for normalizer

To start our analysis, we took advantage of having a collection of muscle biopsies of Becker patients (BMDd45-55) bearing an in-frame deletion of exons 45 to 55 in the *DMD* gene and well characterized from a genetic point of view [22]. This collection has been the subject of a preliminary study which showed notably a decrease in the expression of the protein nNOS in the muscle of the studied patients [22]. We examine here a potential role of miRNA in the regulation of nNOS expression by investigating the expression levels of 617 miRNAs using Taqman Low-Density Array (TLDA) in muscle biopsies of 9 BMDd45-55 patients, compared to 5 control subjects (Table 1). From TLDA data (Additional file 2), we established a list of miRNAs overexpressed in the muscles of BMDd45-55 patients with the criteria of a fold change higher than 2 and a *p* value less than or equal to 0.05. By comparing miRNA expression levels between BMDd45-55 and healthy muscles as control (Fig. 1a), a total of 66 miRNAs were identified based on the defined criteria of fold change and *p* value. Furthermore, the TLDA data were also analyzed by comparing the level of miRNAs expressed in muscles of severe patients with those expressed in muscles of all the other patients (Fig. 1b). This analysis allowed the identification of 29 overexpressed miRNAs. It should be noted that none of these 29 miRNAs were found in the list of miRNAs overexpressed in BMD muscles compared to healthy subjects (Fig. 1a), probably because of the too small number of severe muscle biopsies preventing the fold change value from being statistically significant when included to the values obtained for all the BMD patients.

### miR-708-5p, miR-31-5p, and miR-34c-5p target 3′UTR sequences of *NOS1* gene

**Fig. 2:**
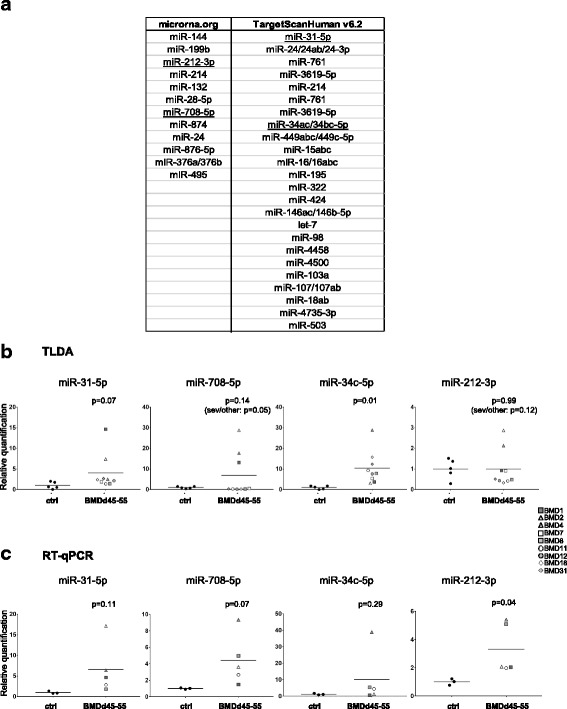
Selection of candidate miRNAs. **a** In silico screening of miRNAs that could target *NOS1* (TargetScan Human and microRNA.org). Candidate miRNAs are underlined. TLDA (Additional file 2, TLDA A2, B2) (**b**) and individual RT-qPCR (**c**) values of candidate miRNA expression in healthy subject biopsies (ctrl, black circle) and BMDd45-55 patients with asymptomatic phenotype (gray circle), moderate phenotype (gray square), severe phenotype (gray triangle), or not determined phenotype (gray hexagon); data are normalized on average of control expression. Lines represent average of each group. Individual RT-qPCR data are expressed as relative quantification using miR-30b as normalizer, normalized on average of control expression

**Fig. 3:**
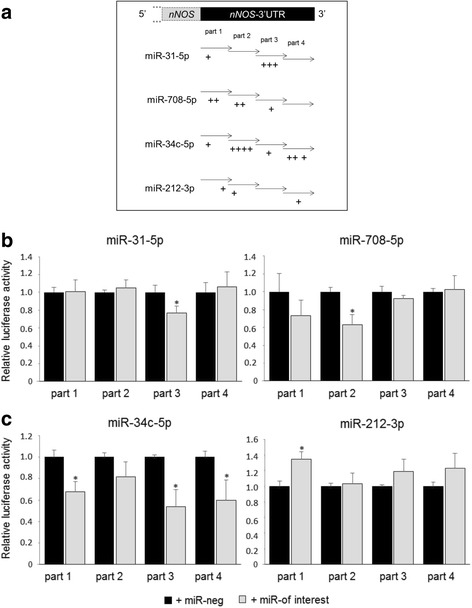
miR-31, miR-708, and miR-34c targeted 3′UTR sequences of *NOS1* gene. **a** Schematic positions of predicted binding sites by microT-CDS Diana Tools in 4 parts of 3′UTR of *NOS1*. **b** Relative luciferase activity of indicated miRNA-transfected cells normalized on luciferase activity in non-specific miRNA transfected cells (miR-neg). Cells were transfected with part 1, part 2, part 3, or part 4 of *NOS1-3′UTR* and with either miR-neg control (black bar) or miR of interest (gray bar). *p < 0.05

To select miRNAs that could modulate nNOS expression, the total sequence of the 3′UTR of the *NOS1* gene (*NOS1*-3′UTR) was submitted to two predictive software, i.e., TargetScan Human and microRNA.org, that process alignment of the target sequence with human miRNA databases (Fig. 2a). From this study, 12 and 24 miRNAs were identified by the 2 predictive software, respectively. Surprisingly, there was no common miRNAs between the two lists. By combining the previous TLDA analysis criteria and the in silico investigation data, 4 miRNAs named miR-31-5p (miR-31), miR-708-5p (miR-708), miR-34c-5p (miR-34c), and miR-212-3p (miR-212) were finally selected. Overexpression of these 4 miRNAs was then validated by performing individual RT-qPCR on 5 BMDd45-55 and 3 healthy muscular biopsies (Fig. 2c). A higher level of expression of the 4 miRNAs was detected in BMDd45-55 compared to control muscles with a fold change of 6.6, 4.4, 10.1, and 3.3 for miR-31, miR-708, miR-34c, and miR-212, respectively, confirming the results obtained by TLDA (Fig. 2b, Additional file 2). Furthermore, by analyzing the sequence of the *NOS1*-3′UTR regarding the 4 selected miRNAs, we identified 5 sequences as potential targets of miR-31, 5 for miR-708, 9 for miR-34c, and 3 for miR-212 (Additional file 3: Table S1 and Fig. 3a). Their ability to bind *NOS1*-3′UTR was then tested in vitro using the luciferase reporter gene. If a miRNA interacted with *NOS1*-3′UTR, we would measure a decreased luciferase signal. Nevertheless, the *NOS1*-3′UTR being 7165 pb in length, it is too large to be fully cloned. Therefore, our strategy was to design 4 sequences (parts #1, #2, #3, and #4) which succeed one another with overlapping avoiding a miRNA-binding sequence being lost and covering all the NOS1-3′UTR sequence (Fig. 3a). Each part was sub-cloned in a plasmid downstream of the luciferase gene, and each of the 4 plasmids was co-transfected in HEK293T cells with one candidate or a non-specific control miRNA mimic. This strategy would also provide a more detailed information about the sequence of *NOS1*-3′UTR implicated in miRNA interaction. Our data showed a significant decrease of luciferase activity when the part #2 was co-transfected with the miR-31 and the part #3 with the miR-708 and when the parts #1, or #3, or #4 were co-transfected with the miR-34c. Nevertheless, no decrease of the reporter gene was observed when miR-212 was co-transfected with the parts #1, #2, #3, nor #4. These results demonstrated that miR31, miR-708, and miR34c, but not miR-212, were able to target *NOS1*-3′UTR sequences leading to a decrease of the reporter gene Firefly luciferase expression.

Altogether, these results demonstrate that these 4 miRNAs were overexpressed in the muscles of BMDd45-55 patients compared to control muscle or in severe patients compared to other patients and that only 3 of them could target sequences present in the *NOS1*-3′UTR and modulate reporter gene activity.

### miR-31, miR-708, and miR-34c effect on nNOS expression in human myoblasts

**Fig. 4:**
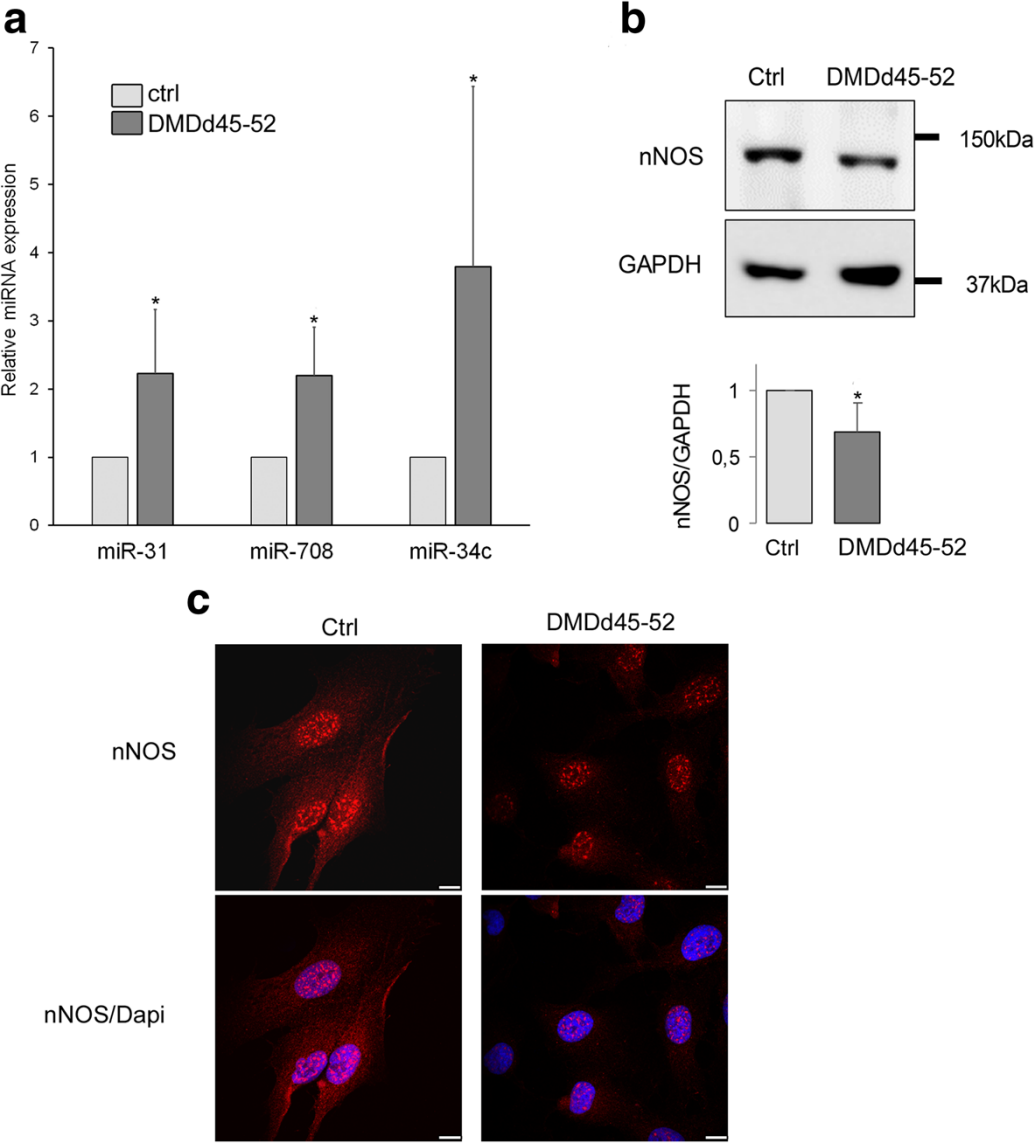
miR-31, miR-708, miR-34c, and nNOS expression in DMDd45-52 myoblasts. **a** miRNA expression in control human myoblasts and DMDd45-52 human myoblasts. Graph represents relative quantification of miRNA normalized on SNORD44 expression. miR-708 *n* = 7, miR-31 *n* = 7, and miR-34c *n* = 8. **b** nNOS immunoblot in control and DMDd45-52 cells. GAPDH serves as the loading control. Bar graph shows quantification results average of 8 independent experiments. **c** Control myoblasts immunolabeled with anti-nNOS (red) antibody, nuclei with Dapi (blue), and imaged by confocal microscopy. Scale bars, 10 μm. Representative of 3 independent experiments. *p ≤ 0.05

In order to address the causal relationship between the overexpression of the 3 selected miRNAs and the nNOS expression in muscular context, we carried out experiments using immortalized human myoblasts from healthy subject (control) and from a patient displaying a deletion of the exons 45 to 52 (DMDd45-52) in the *DMD* gene [29]. First, these DMDd45-52 myoblasts were validated as an appropriate cellular model regarding the expression of the 3 selected miRNAs. Quantification by RT-qPCR confirmed a higher level of expression for miR-31, miR-708, and miR-34c in DMDd45-52 cells compared to control with a fold change of 2.2, 2.2, and 3.8, respectively (Fig. 4a). Furthermore, the expression of nNOS protein was investigated by Western blot and showed a significant decrease in DMDd45-52 compared to control cells (Fig. 4b). Additionally, immunostaining experiments, allowing the detection of the protein nNOS in the cytoplasm and into the nucleus of muscle cells, confirmed that nNOS staining was weaker in the DMDd45-52 compared to the control myoblasts (Fig. 4c). Overall, these results were consistent with those obtained on BMDd45-55 muscle biopsies, namely a higher level of miR-31, miR-708, and miR-34c and a decrease in the expression of nNOS, thus allowing the use of these DMDd45-52 myoblasts as a suitable in vitro cellular model.

**Fig. 5:**
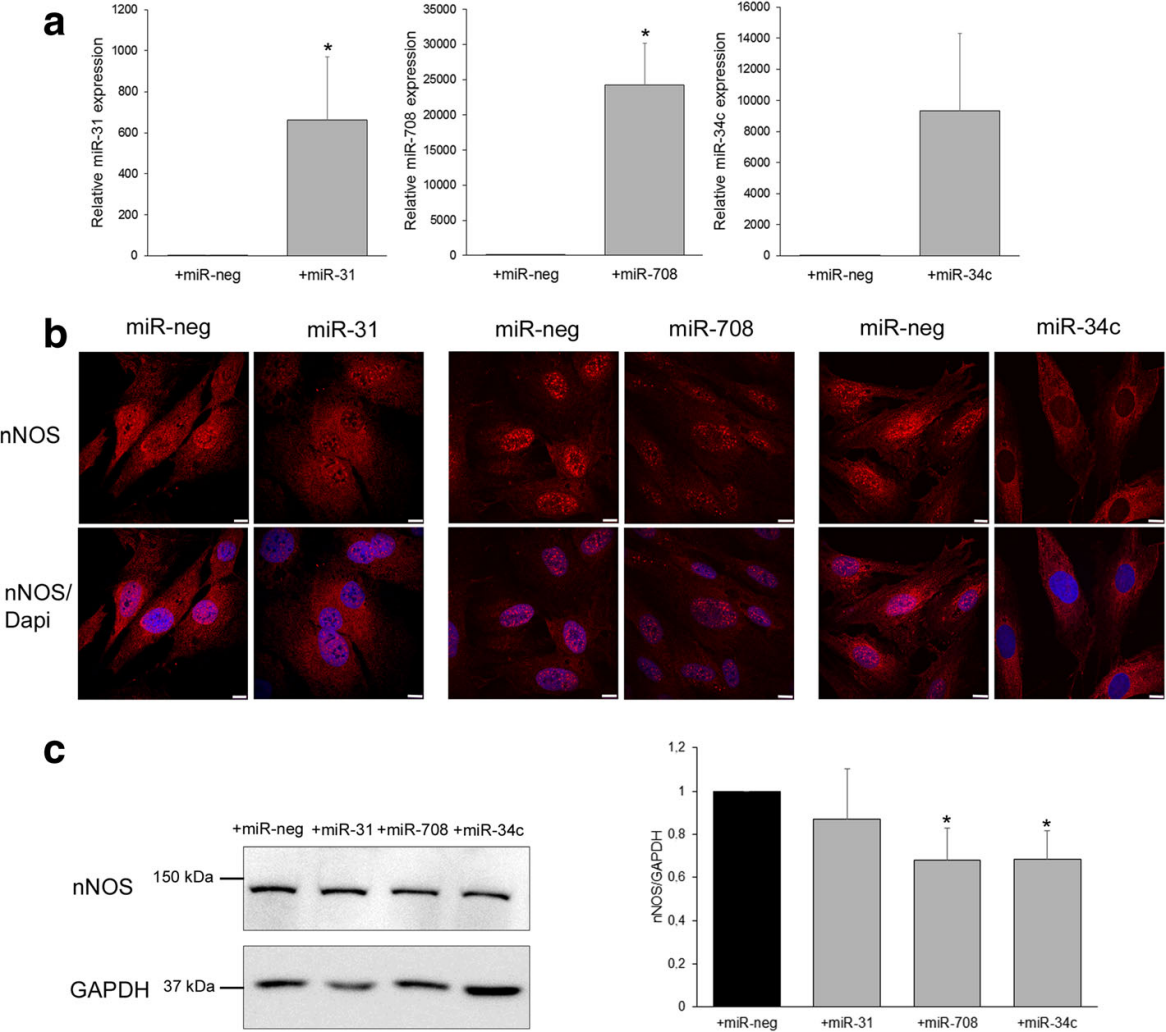
miR-708 and miR-34c overexpression inhibit nNOS expression in transfected control human myoblasts. **a** miRNA expression in control human myoblasts transfected with non-specific control miRNA (miR-neg, black bar) or indicated selected miRNA (gray bar). Graph represents average of relative quantification of miRNA normalized on SNORD44 expression of 5 (miR-31) or 3 (miR-708 and miR-34c) independent experiments. **b** Control myoblasts immunolabeled with anti-nNOS (red) antibody, nuclei with Dapi (blue), and imaged by confocal microscopy. Scale bars, 10 μm. Representative of 4 independent experiments. **c** nNOS immunoblot in transfected control human myoblasts. GAPDH serves as the loading control. Bar graph shows quantification results average of 5 independent experiments. *p ≤ 0.05

To evaluate the effects of the miR-31, miR-708, or miR-34c on the nNOS expression, each of them was transfected in control myoblasts (Fig. 5a). Overexpression of the miRNAs was verified by RT-qPCR (Fig. 5a). The location and expression of nNOS protein were first investigated by immunostaining on the transfected myoblasts (Fig. 5b). Analysis of the pictures showed a decrease of the nNOS labelling in the nuclei of cells overexpressing miR-708 or miR-34c. However, no effect on nNOS expression and location could be observed when miR-31 was overexpressed compared with myoblasts transfected with the non-specific control miRNA. The reduction in the nNOS level was confirmed by Western blot experiments showing a decrease of about 30% of nNOS expression in cells overexpressing miR-708 or miR-34c, while no significant decrease could be observed in overexpressing miR-31 (Fig. 5c). Altogether, these results demonstrated that miR-708 or miR-34c could modulate nNOS expression in human healthy myoblasts.

**Fig. 6:**
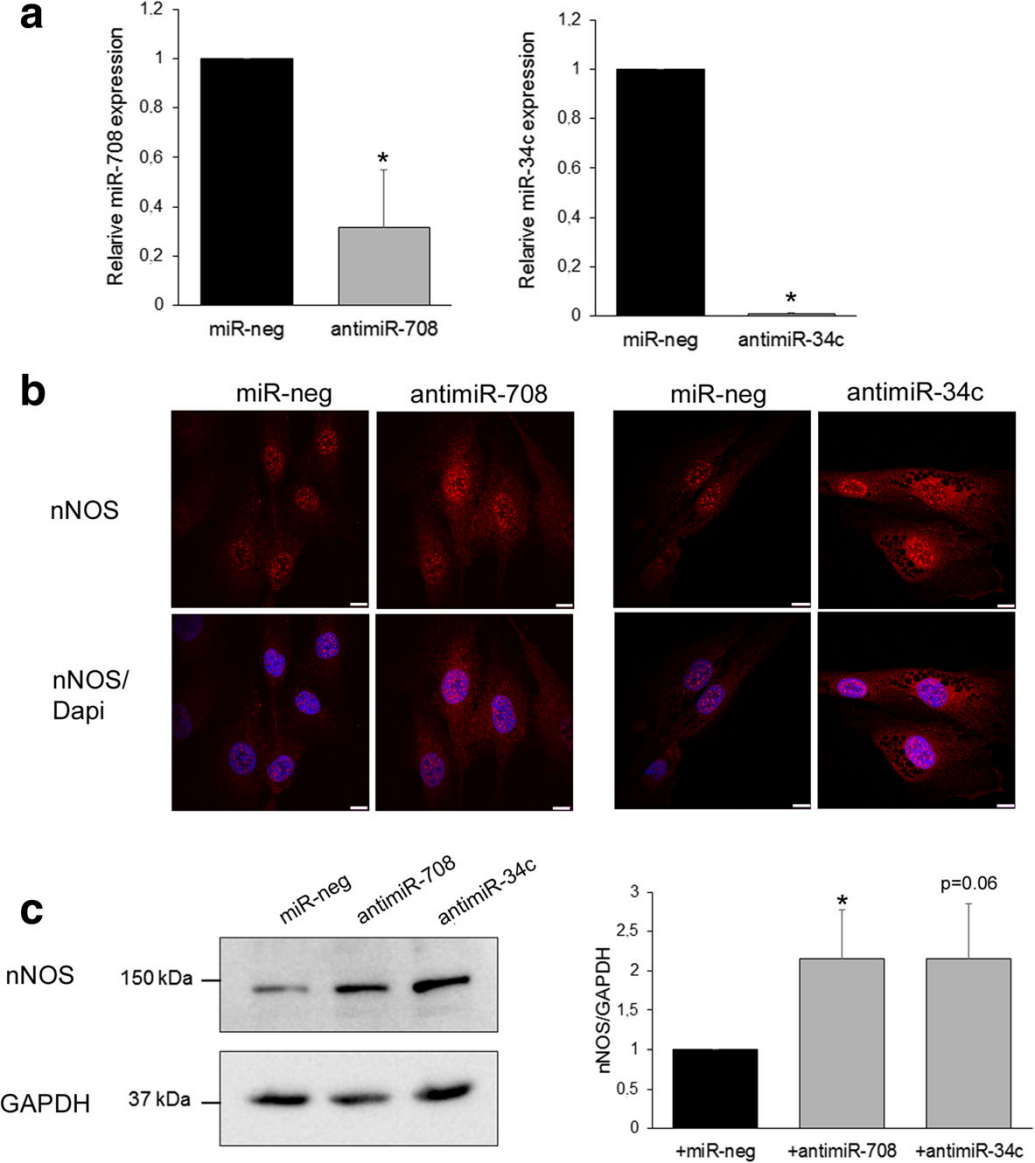
Inhibition of miR-708 and miR-34c increased nNOS expression in transfected DMDd45-52 human myoblasts. **a** miRNA expression in DMDd45-52 human myoblasts transfected with control non-specific miRNA (miR-neg, black bar) or indicated selected antimiR (gray bar). Graph represents average of relative quantification of miRNA normalized on SNORD44 expression of 6 (antimiR-708) or 3 (antimiR-34c) independent experiments. **b** DMDd45-52 myoblasts immunolabeled with anti-nNOS (red) antibody, nuclei with Dapi (blue), and imaged by confocal microscopy. Scale bars, 10 μm. Representative of 5 independent experiments. **c** nNOS immunoblot in transfected DMDd45-52 human myoblasts. GAPDH serves as the loading control. Bar graph shows quantification results average of 5 independent experiments. *p ≤ 0.05

In DMDd45-52 myoblasts, miR-708 and miR-34c expressions increased and nNOS expression decreased compared to control myoblasts (Fig. 4); we thus investigated in these cells the consequences of an inhibition of the miR-708 or the miR-34c by using specific antisense oligonucleotides (antimiR-708 or antimiR-34c) on the nNOS expression level (Fig. 6). The inhibition of miR-708 or the miR-34c levels by their antimiRNAs was validated by RT-qPCR experiments (Fig. 6a). In these cells, the nNOS location and expression were also investigated. Immunofluorescence experiments showed an increased staining in the nuclei of cells in which the miR-708 or the miR-34c were inhibited compared to cells transfected with a non-specific control miRNA (Fig. 6b). These results were confirmed by Western blot experiments that showed a significant increase of 2.2 of nNOS expression in cells transfected with antimiR-708 or antimiR-34c (Fig. 6c).

## Discussion

In this study, we used a variety of bioinformatic, molecular, and cell biological methods to demonstrate the role of miRNAs in driving nNOS expression. We selected 4 miRNAs (i.e., miR-31, miR-708, miR-34c, and miR-212) since they were overexpressed in muscular biopsies of BMDd45-55 patients compared to healthy subjects or in muscular biopsies of patients with severe phenotypes compared to other patients. We then determined, in silico, that these miRNAs could target sequences in *NOS1*-3′UTR. A luciferase reporter study validated the targeting of *NOS1-*3′UTR by miR-31, miR-708, and miR-34c. Finally, we validated the effects of the candidate miRNAs in myoblasts. The experiments were carried out on myoblasts which were a more homogeneous cell population than those of myotubes, from which we never observed 100% of differentiated myotubes and for which efficacy of transfection experiments with miR and antagomiR was much more effective than on differentiated cells. We thus demonstrated that miR-708 and miR-34c could decrease nNOS expression in human healthy myoblasts and that their inhibition led to an increase of this protein in DMDd45-52 human cells.

Several studies showed a deregulation of miRNA expression in muscles of DMD or BMD patients [14, 23, 33] or in serum of DMD patients [26, 34]. Eisenberg et al. studied miRNA profile expression in 10 muscular diseases, and they showed an upregulation of 5 common miRNAs in these diseases [33]. They showed also a particular miRNA expression profile shared by DMD patients and severe BMD patients but not with moderate BMD patients.

Among the selected miRNAs in our study, miR-31 was already shown to be overexpressed in *mdx* mice and in muscular biopsies of DMD patients [14, 23, 35]. We found here the same results in muscular biopsies of BMDd45-55 patients, in DMDd45-52 myoblasts, and in TA muscle of *mdx* mice (data not shown). Unlike our results, Cacchiarelli and colleagues did not observe an increase of miR-31 expression in the biopsies of BMD patients. However, no information on the *DMD* gene mutations and/or phenotypes was given for the patients included in Cacchiarelli et al.’s study. As we found a higher expression of miR-31 in severe phenotypes than in moderate phenotypes (Fig. 2c), we assume that Cacchiarelli et al.’s patients had moderate phenotypes and therefore might not exhibit a high level of miR-31. The fact that miR-31 could target nNOS by mRNA decay was described in human atrial myocytes from patients with atrial fibrillation [36]. In this study, the precise targeted sequence was identified, and it appears to be the same that we identified by the system of cloning *NOS1*-3′UTR downstream luciferase reporter gene setup in our study (Fig. 3). Surprisingly, our data revealed a slight decrease of nNOS expression by miR-31 overexpression in control human myoblasts. One reason could be the level of miR-31. Indeed, in Reilly et al.’s work, miR-31-fold increase was 2 × 10^4^ compared to control condition whereas in our study, miR-31 increased by a factor of 4 × 10^3^ (Fig. 5a) and therefore non-sufficient to exhibit a significant effect. However, we could not transfect more miR-31 because of deleterious effect of transfection on human myoblasts. Nevertheless, one has to consider that the complex regulation displayed by non-coding RNAs might be different according to the studied tissues.

Concerning miR-708 and miR-34c, our results showed an effect of these two miRNAs on nNOS expression in human healthy and DMDd45-52 myoblasts. miR-708 is mostly described in cardiac muscle, where it was proposed to be involved in myocardium regeneration. Indeed, its overexpression in newborn rodents leads to the differentiation of cardiac progenitors to cardiomyocytes by targeting *MAPK14*, a cell cycle gene [37]. Otherwise, miR-708 expression is decreased in murine myoblasts atrophied by dexamethasone treatment, suggesting that miR-708 is involved in muscular development [38]. For miR-34c, several studies described it as overexpressed in *mdx* mice and in DMD patients [23, 35]. Our data were in the same way as miR-34c is overexpressed in BMDd45-55 muscle biopsies, in DMDd45-52 myoblasts, and in *mdx* mice (data not shown). This miRNA was shown to be a promoter of differentiation of murine myoblasts targeting *YY1*, a transcription factor involved in cell proliferation [39] and of porcine satellite cells by inhibiting Notch1 signal pathway that is involved in satellite cell quiescence [40].

The present study revealed that the nNOS expression could be modulated by miR-708 and miR-34c. Our results clearly showed their effect at the protein level, although we did not success to detect nNOS mRNA in myoblasts to demonstrate also the decay of its transcript. Interestingly, it should be noted that the isoform of nNOS that was detected in myoblasts by Western blot is about 140 kDa in size. In mature skeletal muscle, the nNOSμ, a 165-kDa protein, is the major isoform; it is linked to dystrophin via its PDZ domain [41] and thus located mainly at the sarcolemma (Additional file 4: Figure S1). However, this isoform seems too large to correspond to the nNOS isoform detected in myoblasts. Another isoform, the nNOSβ which is 136 kDa in size, not displaying the PDZ domain [42], was described to be present in the Golgi apparatus of skeletal muscle fibers where it modulates the contractile apparatus [17] or at the sarcolemma of mice TA muscles [43]. Western blot experiments on human muscular biopsy of healthy subjects showed a major 160 kDa in size nNOS isoform, as expected but also the 140-kDa isoform (Additional file 4: Figure S1). Additionally, we described here that the nNOS protein was localized in the nuclei of human myoblasts, as shown by immunostaining experiments. Western blots carried out on nuclear and cytoplasmic fractions confirmed that 140 kDa nNOS was detected in nuclei of control and DMDd45-52 myoblasts (Additional file 4: Figure S1). Furthermore, a protein of about 160 kDa in size was only visible in nuclear extracts of both types of cells. These data were compared to those obtained from immunostaining experiments performed on DMD patient muscular biopsies which revealed nNOS expression in the nuclei of fibers of DMD muscle whereas nNOS is sarcolemmal in control muscle as expected (Additional file 4: Figure S1). Nuclear 160-kDa nNOS localization has been already described during C2C12 differentiation; however, authors of this study used a N-terminal nNOS antibody, that did not allow the detection of nNOS-β, and therefore a 140 kDa nNOS isoform [44]. Our data suggest the presence of an isoform of nNOS not yet described in nuclei of myoblasts. At transcriptional level, the precise sequence of a transcript that encodes a nNOS of 140 kDa in size is not described in databases (i.e., Ensembl.org). The complexity of the mechanisms modulating *NOS1* transcription indicates that the nNOS isoform expressed in myoblasts and regulated by miR-34c and miR-708 has not been precisely identified and that information on the transcriptional regulation of its gene remains to be thorough.

The exact role of nNOS in nuclear compartment is still not well-defined. However, NO production has been designated as a key player which mediates epigenetic changes through the direct control of histone deacetylases (HDACs). Indeed, in the *mdx* mice defective for NO pathway, the activity of HDAC2 resulted to be specifically increased [45]. Profiling of human DMDd45-52 patient myoblasts confirmed the dysregulation of miR-1 but also found a significant dysregulation in the expression of miR-29a, both of which regulate a dystrophin-nNOS-HDAC2 pathway [14]. In the present study, we could not exclude a link between nuclear nNOS location, HDAC2 nitrosylation, and the modulation of the miR-31, miR-708, and/or miR-34c expression. Nevertheless, a study in its own right will be necessary to establish this link.

## Conclusions

Altogether, the present work highlights two miRNAs overexpressed in dystrophic human muscle as modulators of nNOS expression. This work could explain some pathological consequences caused by nNOS deficiency (i.e., muscle fatigability due to insufficient vasodilation in exercise, switch to glycolytic metabolism). In particular, modification of NOS1 expression has a significant negative impact on dystrophic muscle regenerative capacity [15], and it has been shown that treatment with NO donors can attenuate atrophy and metabolic changes and prevent changes in regulation [16]. We show here that inhibitors of miR-708 and/or miR-34c could also be considered as therapeutic targets to rescue these defects by increasing the expression of nNOS. Furthermore, the expression and the sarcolemmal localization of the nNOS by interacting with the dystrophin has been shown to be crucial for contractile activity and muscular strength recovery in the canine DMD model (GRMD) [46]. Thus, a therapeutic strategy combining the inhibition of miR-708 and miR-34c with the restoration of dystrophin will most likely be a benefit for the improvement of phenotype of DMD and BMD patients.

## Additional files

Additional file 1: Supplementary methods. (DOCX 12 kb)

Additional file 2: TLDA data. (XLSX 749 kb)

Additional file 3: **Table S1.** Predictive candidate miRNA binding sites on the human NOS1 3’UTR (DOCX 12 kb)

Additional file 4: **Figure S1.** Nuclear localization of nNOS in DMD muscular biopsy and in myoblasts. a) Control (ctrl) and DMD human muscular biopsy sections immunolabeled with anti-nNOS (red) antibody, nuclei with Dapi (blue), and imaged by confocal microscopy. Representative of 4 DMD patients. b) nNOS, GAPDH, and histone H3 (H3) immunoblots on cytoplasmic (CE) and nuclear (NE) protein extracts from control (ctrl) and DMDd45-52 myoblasts and total extract of control human muscular biopsy (ctrl biopsy). (TIFF 912 kb)

## Abbreviations

BMD: Becker muscular dystrophy
DAPC: Dystrophin-associated protein complex
DMD: Duchenne muscular dystrophy
GMWS: Gardner–Medwin and Walton scale
GRMD: Golden Retriever muscular dystrophy
HDAC: Histone deacetylase
nNOS: Neuronal nitric oxide synthase
TLDA: Taqman Low-Density Array
