# Supplementary material for: miR-708-5p and miR-34c-5p are involved in nNOS regulation in dystrophic context
Source: Skelet Muscle. 2018 Apr 27;8:15. doi: 10.1186/s13395-018-0161-2 (PMC5924477; doi:10.1186/s13395-018-0161-2)
Supplement: Supplementary file 1 — Supplementary methods. (DOCX 12 kb) [file 13395_2018_161_MOESM1_ESM.docx]

**Supplementary methods**

**Nuclear and Cytoplasmic protein extraction.**

Cells were harvested in trypsin and nuclear and cytoplasmic proteins were extracted with NE-PER™ Nuclear and Cytoplasmic Extraction Reagents (Thermofisher scientific) according to manufacturer’s instructions. Western-blotting were performed as described in Material and Methods.

**Immunostaining of human muscular biopsies sections.**

Sections were cut at 8 µm on a cryostat, permeabilized by 0.5% Triton X-100 and blocked in phosphate-buffered saline (PBS)- 4% bovine serum albumin (BSA) for 1 hr. Sections were incubated in PBS-4% BSA-0.1% Triton X-100 with primary goat polyclonal anti nNOS antibody (Abcam) overnight at room temperature and washed in PBS. Sections were then incubated with secondary antibody for 1 hr, washed in PBS, incubated with DAPI for nuclear staining, and mounted in Fluoromount-G (Clinisciences). Images were acquired or Leica SPE confocal microscope.
