# Supplementary material for: miR-708-5p and miR-34c-5p are involved in nNOS regulation in dystrophic context
Source: Skelet Muscle. 2018 Apr 27;8:15. doi: 10.1186/s13395-018-0161-2 (PMC5924477; doi:10.1186/s13395-018-0161-2)
Supplement: Supplementary file 3 — Table S1. Predictive candidate miRNA binding sites on the human NOS1 3’UTR (DOCX 12 kb) [file 13395_2018_161_MOESM3_ESM.docx]

**Additional file 3: Table S1: predictive candidate-miRNAs binding sites on the human NOS1 3'UTR**

| **miRNA** | **position in *NOS1* 3'UTR** |
| --- | --- |
| **212-3p** | 1729-1757 |
|  | 2066-2094 |
|  | 6700-6728 |
| **708-5p** | 152-180 |
|  | 382-410 |
|  | 1982-2010 |
|  | 2191-2219 |
|  | 5167-5195 |
| **31-5p** | 232-260 |
|  | 4349-4377 |
|  | 4593-4621 |
|  | 4728-4756 |
|  | 6156-6184 |
| **34c-5p** | 67-95 |
|  | 1844-1872 |
|  | 2208-2236 |
|  | 3286-3314 |
|  | 3313-3341 |
|  | 4882-4910 |
|  | 5368-5396 |
|  | 5478-5506 |
|  | 5488-5516 |
|  | 5778-5806 |
| **376b** | 1759 |
