# Supplementary figures and images for: miR-708-5p and miR-34c-5p are involved in nNOS regulation in dystrophic context
Source: Skelet Muscle. 2018 Apr 27;8:15. doi: 10.1186/s13395-018-0161-2 (PMC5924477; doi:10.1186/s13395-018-0161-2)

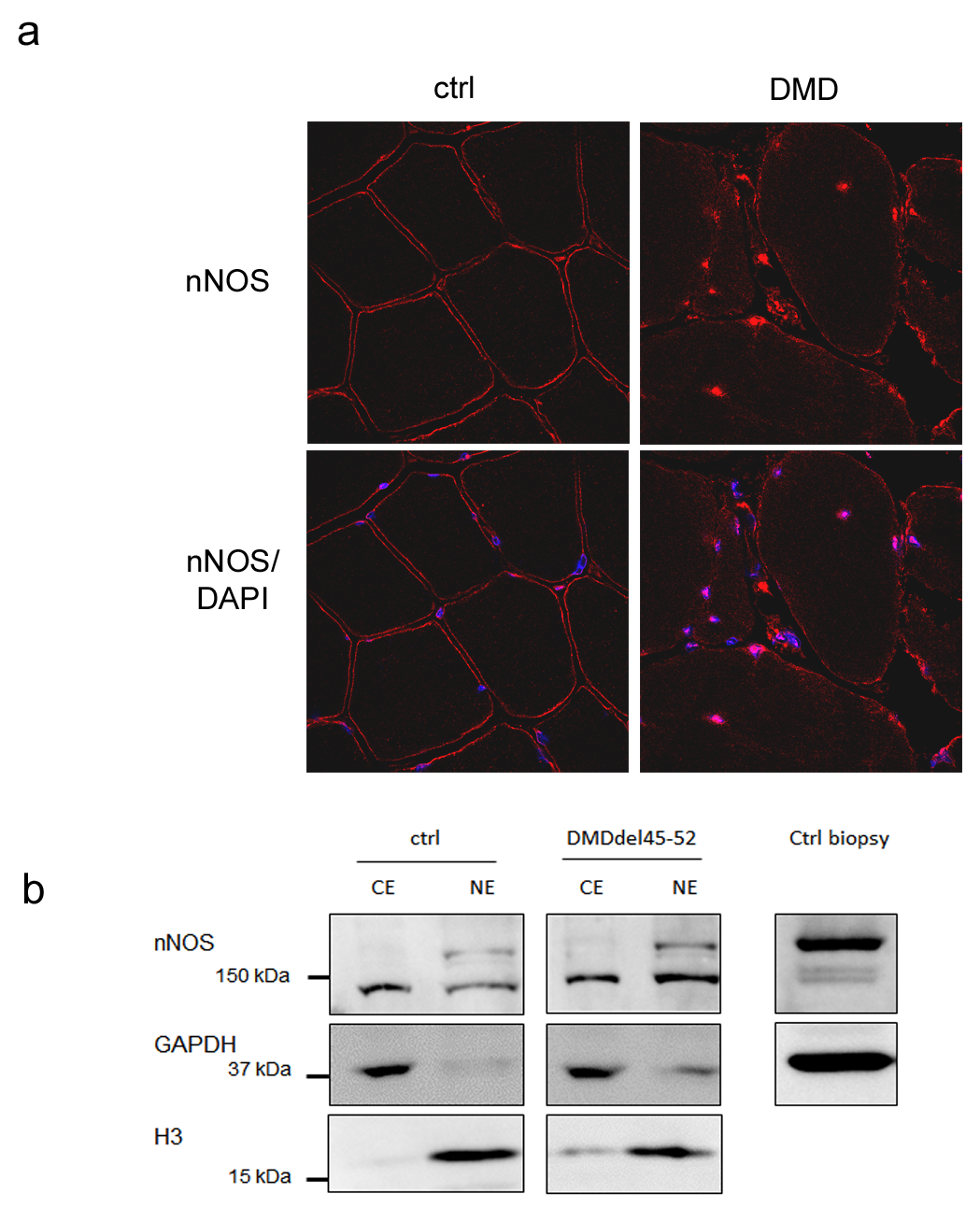

Supplement: Supplementary file 4 — Figure S1. Nuclear localization of nNOS in DMD muscular biopsy and in myoblasts. a) Control (ctrl) and DMD human muscular biopsy sections immunolabeled with anti-nNOS (red) antibody, nuclei with Dapi (blue), and imaged by confocal microscopy. Representative of 4 DMD patients. b) nNOS, GAPDH, and histone H3 (H3) immunoblots on cytoplasmic (CE) and nuclear (NE) protein extracts from control (ctrl) and DMDd45-52 myoblasts and total extract of control human muscular biopsy (ctrl biopsy). (TIFF 912 kb) [file 13395_2018_161_MOESM4_ESM.tif]
